# Telomere tracking from birth to adulthood and residential traffic exposure

**DOI:** 10.1186/s12916-017-0964-8

**Published:** 2017-11-21

**Authors:** Esmée M. Bijnens, Maurice P. Zeegers, Catherine Derom, Dries S. Martens, Marij Gielen, Geja J. Hageman, Michelle Plusquin, Evert Thiery, Robert Vlietinck, Tim S. Nawrot

**Affiliations:** 1 Centre for Environmental Sciences, Hasselt University, Agoralaan Building D, 3590 Diepenbeek, Belgium; 2 Department of Complex Genetics, NUTRIM School of Nutrition and Translational Research in Metabolism, Maastricht University Medical Centre, Maastricht, The Netherlands; 3 CAPHRI School for Public Health and Primary Care, Maastricht University, Maastricht, The Netherlands; 4 Department of Obstetrics and Gynecology, Ghent University Hospitals, Ghent, Belgium; 5 Centre of Human Genetics, University Hospitals Leuven, Leuven, Belgium; 6 Department of Toxicology, NUTRIM School of Nutrition and Translational Research in Metabolism, Maastricht University Medical Centre, Maastricht, The Netherlands; 7 Department of Neurology, Ghent University Hospitals, Ghent, Belgium; 8 Department of Public Health, Leuven University (KU Leuven), Leuven, Belgium

**Keywords:** Telomere length, Traffic, Tracking

## Abstract

**Background:**

Telomere attrition is extremely rapid during the first years of life, while lifestyle during adulthood exerts a minor impact. This suggests that early life is an important period in the determination of telomere length. We investigated the importance of the early-life environment on both telomere tracking and adult telomere length.

**Methods:**

Among 184 twins of the East Flanders Prospective Twin Survey, telomere length in placental tissue and in buccal cells in young adulthood was measured. Residential addresses at birth and in young adulthood were geocoded and residential traffic and greenness exposure was determined.

**Results:**

We investigated individual telomere tracking from birth over a 20 year period (mean age (SD), 22.6 (3.1) years) in association with residential exposure to traffic and greenness. Telomere length in placental tissue and in buccal cells in young adulthood correlated positively (r = 0.31, *P* < 0.0001). Persons with higher placental telomere length at birth were more likely to have a stronger downward shift in telomere ranking over life (*P* < 0.0001). Maternal residential traffic exposure correlated inversely with telomere length at birth. Independent of birth placental telomere length, telomere ranking between birth and young adulthood was negatively and significantly associated with residential traffic exposure at the birth address, while traffic exposure at the residential address at adult age was not associated with telomere length.

**Conclusions:**

Longitudinal evidence of telomere length tracking from birth to adulthood shows inverse associations of residential traffic exposure in association with telomere length at birth as well as accelerated telomere shortening in the first two decades of life.

**Electronic supplementary material:**

The online version of this article (doi:10.1186/s12916-017-0964-8) contains supplementary material, which is available to authorized users.

## Background

Telomeres are located at the end of chromosomes and protect these regions from degradation [1]. Most human somatic tissues are not able to maintain telomere length, with telomeres shortening during cell division and resulting in telomere attrition with cellular age [2]. Eventually, telomeres reach a critical length, leading to loss of protection and chromosomal instability [3]. Short telomeres have been implicated in the pathology of several age-related diseases, including cardiovascular disease [4], diabetes mellitus [5], and cancer [6, 7]. Moreover, an association between telomere length and mortality has been observed in studies of elderly twins, showing that the twin with the shortest telomere length has a greater risk of death during follow-up compared to the co-twin with the longest telomeres [8, 9]. These results indicate that telomere length functions as a measure of biological aging [10, 11].

The decline of telomere length with age has been observed in longitudinal studies starting from early adult life until advanced age [12–14]. However, cross-sectional studies comparing individuals with a wide age range, from neonates to the elderly, observed that the rate of telomere attrition varied at different ages [15, 16]. Further, in more than 500 individuals, aged from 0 to 90 years, telomere loss was more pronounced in the first year and continued thereafter at a slower rate [16]. In addition, a longitudinal study measuring telomere length in samples donated 12 years apart by 1156 participants observed that, in adults, telomere length ranking does not change much over time [17]. These results suggest that most of the inter-individual variation in telomere length among adults is established early in life and that lifestyle during adulthood exerts only a minor impact on telomere ranking [17]. Thus, early life is an important period in the determination of telomere length and could have a lasting effect on telomere length throughout the life course [18].

Telomere length has been associated with exposure to different types of air pollution [11, 19–21]. Shorter leukocyte telomere length was observed in traffic officers compared to office workers [20] and long-term exposure to airborne particles, especially those related to traffic, is associated with faster telomere attrition in 70-year-olds [19].

Compared with term-born singletons, twins are more susceptible to prenatal exposure of particulate air pollution [22]. In addition, we observed in previous research that maternal residential proximity to traffic and lower residential surrounding greenness is associated with shorter placental telomere length at birth in twins [23]. Currently, there is a gap in the understanding of the importance of the early-life environment on both telomere tracking and adult life telomere length. Herein, we measured telomere length at birth in placental tissue and in young adulthood in buccal cells to determine if early-life exposure to traffic has long-term consequences on telomere length. Further, we studied the effects of traffic exposure early in life on telomere tracking over the first two decades of life.

## Methods

### Subjects

**Fig. 1:**
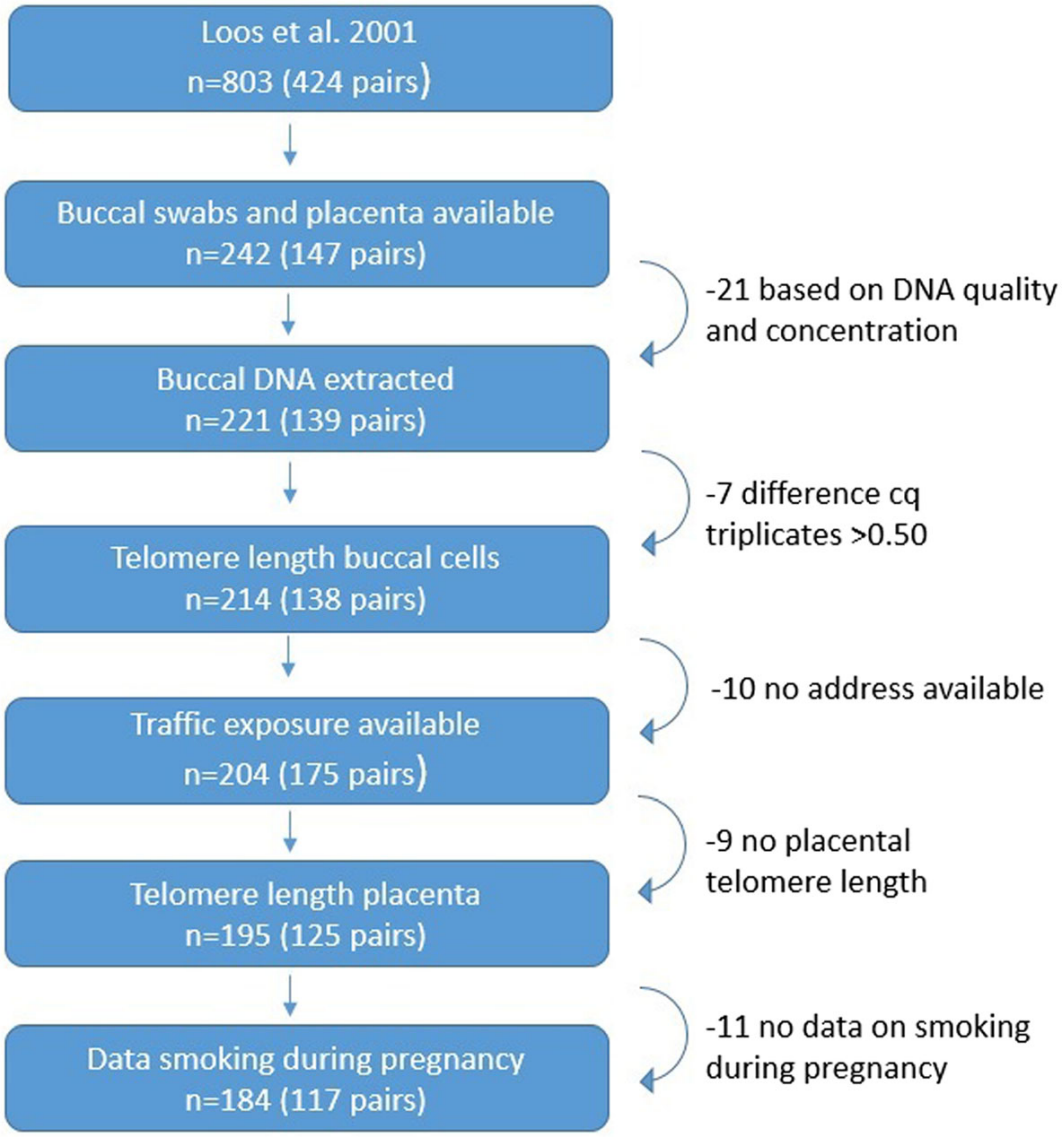
The present study sample consisted of 233 twins of Caucasian origin (99% naturally conceived), born between 1969 and 1982, who participated in a prenatal programming study and had both placental telomere length and buccal swabs at adulthood available. We excluded 49 participants from our analysis (1) because DNA quality or concentration was insufficient (n = 21) or because triplicate measurements of telomere length were too variable (difference in quantification cycle more than 0.50) (n = 7), (2) because residential address was missing (n = 10), or (3) because information on smoking status during pregnancy was not filled out in the questionnaire (n = 11). The number of twins included in our analysis was 184

The East Flanders Prospective Twin Survey, a population-based register of multiple births in the province of East Flanders (Belgium), commenced enrollment of twins at birth in 1964 [24]. The twin-population in our study was based on a previous twin study by Loos et al. [25]. Young adult twins aged 18–34 years (803 pairs) were randomly contacted through an envelope system. Eventually, 424 pairs (overall response of 52.8%) agreed to participate [25]. The present study sample consisted of 233 twins of Caucasian origin (99% naturally conceived), born between 1969 and 1982, who participated in a prenatal programming study and had both placental telomere length and buccal swabs at adulthood available. We excluded 49 participants from our analysis (1) because DNA quality or concentration was insufficient (n = 21) or because triplicate measurements of telomere length were too variable (difference in quantification cycle more than 0.50) (n = 7), (2) because residential address was missing (n = 10), or (3) because information on smoking status during pregnancy was not provided in the questionnaire (n = 11). The number of twins included in our analysis was 184. The dropout rate of the present study is described in the flowchart (Fig. 1).

### Telomere length measurements in placental tissue

All placental biopsies were taken from the maternal side of the placenta, 2 cm from the umbilical cord insertion site, free of maternal decidua and stored at –20 °C. Prior to DNA isolation the placental tissue was washed with phosphate-buffered saline to remove possible traces of maternal blood. DNA was isolated from placental tissue using the QIAamp DNeasy blood and tissue kit (Qiagen, Venlo, The Netherlands), following the manufacturer’s instructions for animal tissues. Quality and concentration of the isolated placental DNA were assessed using the Nanodrop 1000 spectrophotometer (Isogen Life Science, Belgium). Twin pairs were excluded from the analyses when DNA samples had a low yield or to absorption ratios for A260/A280 that were outside the range of 1.8 to 2.0. The methods for telomere length measurement in placental tissue have been previously described [23, 26]. In brief, telomere length was determined using a monochrome multiplex quantitative PCR (q-PCR) method [27]. For multiplex q-PCR, telomere primer pairs telg (at 900 nm; ACACTAAGGTTTGGGTTTGGGTTTGGGTTTGGGTTAGTGT) and telc (at 900 nm; TGTTAGGTATCCCTATCCCTATCCCTATCCCTATCCCTAACA) were combined with the beta-globin primer pairs hbgu (at 500 mM; CGGCGGCGGGCGGCGCGGGCTGGGCGGcttcatccacgttcaccttg) and hbgd (at 500 mM; GCCCGGCCCGCCGCGCCCGTCCCGCCGgaggagaagtctgccgtt). Reference genomic DNA (Hela 229 cell line, telomere length of 14.5 kbp) was used to generate two standard curves for each PCR plate, one for the telomere signal and one for the single copy gene signal to determine primer efficiency. Samples were assayed in triplicate and the average was used. Based on the reference samples, the coefficient of variation was 2.5% for within plate measurements and 4.9% for measurements between plates. The inter-assay coefficient of variation was 13.03%. We observed no correlation between the coefficient of variation with mean placental telomere length of the triplicates (r = 0.06; *P* = 0.40).

### Telomere length measurements in buccal cells

Mouth swabs were taken and DNA was extracted with the QIAamp DNA micro kit (Qiagen, Venlo, The Netherlands). DNA purity and concentration was assessed using the Nanodrop 1000 spectrophotometer (Isogen Life Science, Belgium). Due to a low DNA yield or to absorption ratios for A260/A280 that were outside the range of 1.8 to 2.0 for buccal DNA, 21 individuals were excluded from the analyses. Relative telomere length was measured in buccal swabs using an adapted quantitative real-time PCR method [27]. Relative telomere length is based on the ratio of telomere sequence repeats to a single copy nuclear control gene, *36B4* (acidic ribosomal phosphoprotein P0) [27]. Extracted genomic DNA was diluted, resulting in a final input of 5 ng. The telomere PCR reaction mixture contained 1X QuantiTect SYBR® Green (Qiagen, Venlo, The Netherlands) Mastermix, 300 nM primer telg (ACACTAAGGTTTGGGTTTGGGTTTGGGTTTGGGTTAGTGT), 900 nM primer telc (TGTTAGGTATCCCTATCCCTATCCCTATCCCTATCCCTAACA), and 2.5 mM DTT. The single copy gene PCR reaction mixture contained 1X QuantiTect SYBR® Green (Qiagen, Venlo, The Netherlands) Mastermix, 300 nM forward (CAGCAAGTGGGAAGGTGTAATCC), and 500 nM reverse primer (CCCATTCTATCATCAACGGGTACAA).

Each PCR reaction was performed in triplicate and three non-template controls as well as six inter-run calibrators were included on each 384-well plate. All samples were analyzed using a 7900HT Fast Real-Time PCR system (Life Technologies). Telomere PCR conditions were one cycle at 95 °C for 10 min followed by two cycles of 15 sec at 94 °C and 2 min at 49 °C, and 30 cycles of 15 sec at 94 °C, 20 sec at 62 °C, and 1 min 40 sec at 74 °C. Single copy gene PCR conditions were one cycle at 95 °C for 10 min followed by 40 cycles of 15 sec at 95 °C and 1 min 10 sec at 58 °C.

After thermal cycling, raw data were collected and processed. The relative average telomere length was calculated as the ratio of the cycle threshold value of telomere sequence repeat to single copy gene (T/S) in the study subjects compared with that of the averaged T/S value for the study population using the qBase software (Biogazelle, Zwijnaarde, Belgium). This program uses modified software from the classic comparative cycle threshold method and uses inter-run calibration algorithms to correct for run-to-run differences [28]. All samples were analyzed in triplicate and included in the study when the difference in quantification cycle value was less than 0.50. The coefficients of variation within triplicates of the telomere and single copy gene assay were 0.48% and 0.31%, respectively. The inter-assay coefficient of variation was 7.38%. We observed no correlation between the coefficient of variation with the mean buccal telomere length of the triplicates (r = 0.10; *P* = 0.17).

### Data collection

Data recorded by the obstetrician at birth included gestational age, birth weight, sex of the twins, and parental ages. Gestational age was based on the last menstruation and was calculated as the number of completed weeks of pregnancy. Zygosity was determined at birth by sequential analysis based on sex, choriontype, blood group determined on umbilical cord blood, placental alkaline phosphatase and, since 1982, DNA fingerprints [29]. After DNA-fingerprinting, a zygosity probability of 99.9% was reached.

At a later stage, the parents of the twins filled out questionnaires. In this way, maternal smoking during pregnancy and parental educations was collected retrospectively. Educational level as a proxy of socioeconomic status was categorized into three groups according to the Belgian education system, namely as no education or primary school, lower secondary education, and higher secondary education and tertiary education. The twins completed questionnaires to obtain information on smoking status.

Biometric and laboratory measurements of adult twins were obtained at the research center during a 2-h morning session. Body mass index was calculated as body mass (in kg) divided by squared height (in m). Total cortisol was determined in 24-h urine using the ADVIA Centaur Cortisol assay. In fasting blood samples, gamma-glutamyl transferase was measured on an Olympus AU600 Auto-Analyzer (Kyoto, Japan). We use gamma-glutamyl transferase as a marker of alcohol intake [30]. Cortisol, which is synthesized from cholesterol, is a biochemical marker of chronic stress [31].

### Traffic exposure

Residential addresses of the mothers at time of birth of the twins and the residential addresses of the twins at time of the measurement were geocoded. Residential distances to the nearest major road was determined using Geographic Information System functions. Major roads were defined by two types of roads, namely freeways and national roads. All Geographic Information System analyses were performed using ArcGIS 10 software.

### Statistical analysis

For data management and statistical analyses, we used SAS software, version 9.4 (SAS Institute, Cary, NC). All reported *P* values are two-sided and were considered statistically significant when *P* < 0.05. The distribution of all variables was inspected and the measure of distance from residence to major road was log-transformed because this parameter had a skewed distribution and also because traffic-related pollutants decay exponentially with increasing distance from roads [32, 33].

Because telomere measurements were measured in different tissues at baseline (placenta) and follow-up (buccal cells), we used a ranking approach to study telomere tracking over a median follow-up of 22.6 years (range, 18.0–29.8). First, we ranked relative telomere length from long to short at birth (placental) and at young adulthood (buccal). The newborn with the longest telomere was coded as 1 and the newborn with the shortest was coded as 184, the same procedure was applied for adult telomere length. Subsequently, we calculated the difference between the telomere rank for each individual by subtracting the rank at adulthood from the rank at birth. Because this difference in ranking depends also on the number of subjects in the population, we converted this to obtain a value with a maximum of 100 to facilitate interpretation. A positive value indicates an increase in ranking and a negative value a decrease in ranking from birth to young adulthood as a relative change between telomere length at birth and young adulthood compared with other individuals of the study population.

The difference in telomere ranking (ΔR) between birth and adulthood was calculated as follows:

$$ \varDelta R=\frac{\left({R}_1-{R}_2\right)}{\left(\frac{n-1}{100}\right)} $$

Where n is the number of subjects in study population, R1 is the rank (from 1 to n) based on telomere length at birth, and R2 is the rank (from 1 to n) based on telomere length in adulthood.

Mixed modeling was performed to investigate buccal telomere length in young adulthood and telomere tracking in association with covariates. To differentiate between early-life and adulthood exposure to traffic and greenness, we performed stratified analysis for twins who were living at the same address their whole life (non-movers, n = 62) and twins who were living at a different address than their birth address at the time of sample collection (movers, n = 122) [34].

The twins were analyzed as individuals in a multilevel regression analysis to account for relatedness between twin members by adding a random intercept to the model. The variance-covariance structure was allowed to differ between the three zygosity and chorionicity groups, including dizygotic dichorionic, monozygotic dichorionic, and monozygotic monochorionic. Mixed modeling was performed adjusted for covariates selected a priori, namely newborn sex, birth weight, gestational age, zygosity and chorionicity, parental educational level, maternal smoking during pregnancy, and maternal age. In addition, at birth, we adjusted for birth year and, in models including telomere length at adulthood and telomere ranking, we included adult age, smoking in adulthood, and gamma-glutamyl transferase in fasting blood in adulthood (as an index for alcohol consumption). To account for the phenomenon of regression towards the mean, we included telomere length at birth in longitudinal analysis (ranking) and we tested the interaction between baseline telomere length and our exposure variable (residential distance to major road). The interaction term between baseline telomere length (birth) and early-life traffic exposure was not significant (*P* = 0.26) and therefore not kept in the model.

We ran two sensitivity analyses, namely (1) with additional adjustment for 24-h urinary cortisol in adulthood and (2) combining both the early-life and adulthood residential distance to a major road.

## Results

### Characteristics of the study population

Table 1 summarizes the characteristics of the study population. The study population includes 117 mothers; 57.3% (n = 134) of the participants included both twins from each twin pair, whereas the remaining 42.7% (n = 50) only had one participating twin from each twin pair. Information on telomere length in buccal cells and placental tissue was available for 184 twins, of which 121 (65.8%) were monozygotic and 63 (34.2%) dizygotic. At the time of collection of buccal swabs the twins had a mean (SD) age of 22.6 (3.1) years. The median distance to the nearest major road was 352 m (interquartile range (IQR), 166–1218 m) at the residential address at birth and 392 m (IQR, 128–822 m) at the residential address at adult age.

**Table 1:** Study population characteristics

Maternal	n = 117
Maternal age, years	26.7 ± 4.1
Smoking during pregnancy, n	13 (11.1)
Socioeconomic status: maternal education	
Low	46 (39.3)
Middle	28 (23.9)
High	43 (36.8)
Birth	n = 184
Gestational age, weeks	37.2 ± 2.5
Neonate birth weight, g	2589 ± 506
Twin birth year	1976 ± 3.2
Zygosity – Chorionicity	
Dizygotic – Dichorionic	63 (34.2)
Monozygotic – Dichorionic	55 (29.9)
Monozygotic – Monochorionic	66 (35.9)
Adulthood	n = 184
Age, years	22.6 ± 3.1
Sex	
Male	93 (50.5)
Female	91 (49.5)
Body mass index, kg/m^2^	21.5 ± 2.7
Smokers, n	64 (34.8)
Gamma-glutamyl transferase, U/L	17.8 ± 11.1
Total cortisol (μg/dL)	97.1 ± 41.4
Complete-pair in final study	
One twin	50 (42.7)
Both twins	134 (57.3)
Moved since birth	122 (66.3)

Data presented are means ± standard deviation or number (percentage)

**Table 2:** Predictors of telomere length ranking between birth and young adulthood

	Multi variable model		
Predictors	Change in ranking	95% CI	*P* value
Early-life covariates			
Placental telomere length, + IQR	–24.6	–29.4 to –19.9	<0.0001
Newborn girls	9.9	0.8 to 19.0	0.04
Birth weight, +IQR	1.3	–6.4 to 9.0	0.76
Gestational age, +IQR	–3.4	–10.2 to 3.4	0.34
Parental education level	–5.4	–10.5 to –0.4	0.04
Smoking during pregnancy	–8.3	–23.0 to 6.4	0.27
Residential distance to major road early-life, 2-fold change	8.7	1.2 to 16.1	0.03
Adult covariates			
Age, +1 year	–1.7	–3.2 to –0.3	0.03
Smoking	–8.3	–17.1 to 0.5	0.07
Gamma-glutamyl transferase, +IQR	–4.0	–7.4 to –0.7	0.02

To examine telomere tracking over life we ranked telomere length at birth (based on placental telomere) and at adult life (based on buccal cell telomere length). We studied the difference in ranking, a negative difference in ranking between birth and young adulthood means a decline in ranking to 100 faster than the average of the population

*CI* confidence interval, *IQR* interquartile range

### Association between telomere length in placental tissue and buccal swabs

**Fig. 2:**
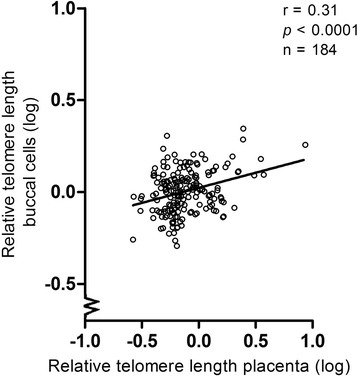
Relative telomere length in buccal cells in young adulthood in association with relative placental telomere length at birth

Telomere length in buccal cells in adulthood was positively associated with placental telomere length (r = 0.31, *P* < 0.0001) (Fig. 2). The mean T/S (SD) was 0.90 (0.78) for placental tissue and 1.05 (0.29) in adult buccal cells.

### Determinants of telomere tracking from birth to adulthood

Participants with a relative long telomere length at birth exhibited a stronger downward (accelerated shortening) shift in ranking compared to participants with short placental telomeres (–24.6; 95% confidence interval (CI) –29.4 to –19.9; *P* < 0.0001). In addition, a downward shift in telomere ranking was also associated with higher adult age (*P* = 0.03), higher alcohol intake (*P* = 0.02), and higher parental education (*P* = 0.04). An upward shift (decelerate shortening) in telomere ranking between birth and adulthood was observed in women compared with men (*P* = 0.04). Table 2 shows these results.

### Birth and adulthood telomere length and residential traffic indicators

**Fig. 3:**
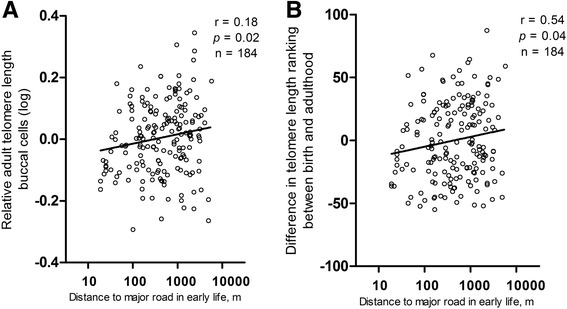
**a** Relative telomere length in buccal cells in adulthood (log) in association with distance to major road at the residential address at birth. **b** Difference in telomere length ranking between birth and adulthood adjusted for telomere length in placental tissue is associated with distance to major road at the residential address at birth

**Fig. 4:**
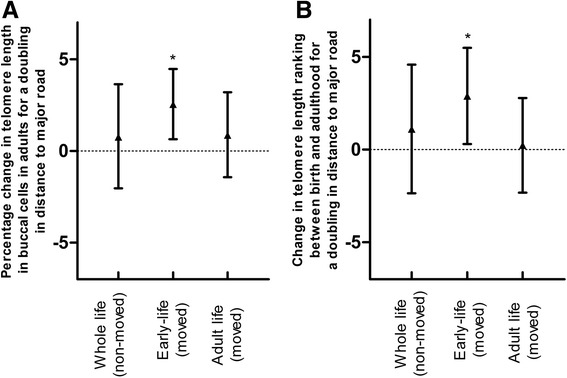
Distance to the nearest major road in association with (**a**) telomere length in buccal cells and (**b**) change in telomere length ranking between birth and adulthood. Adjusted for newborn sex, birth weight, gestational age, zygosity and chorionicity, parental educational level, maternal smoking during pregnancy, maternal age, adult age, smoking in adulthood, gamma-glutamyl transferase in fasting blood in adulthood (as an index for alcohol consumption), and telomere length in placental tissue at birth. Vertical lines denote 95% confidence intervals. *Indicates a significant (*P* < 0.05) change in buccal telomere length in adulthood or a change in telomere ranking. Effect size for a two-fold increase in distance from residence to major road in early/adult life (based on a model with log distance) in movers (n = 122) or in distance from residence to major road during whole life in non-movers (n = 62)

Twins living farther away from a major road at the birth address had both longer placental telomere at birth and longer buccal telomere length at adulthood. In twins who moved during life (n = 122), the corresponding estimates expressed for a doubling in distance between residence and major road showed 3.6% longer placental telomeres at birth (95% CI 0.3 to 7.0; *P* = 0.04) and a 2.5% longer buccal telomere length in adulthood (95% CI 0.6 to 4.5; *P* = 0.01). This association was observed before (Fig. 3a) and after adjustments for covariates (Fig. 4a). After additional adjustment for 24-h urinary cortisol (adulthood), the association between buccal telomere length and traffic exposure remained significant (Additional file 1: Figure S1A).

### Telomere tracking from birth to adulthood and residential traffic

We noted that traffic exposure in early life is significantly associated with telomere length in adulthood and with telomere ranking in twins who moved during life (living at a different address than their birth address at the time of measurement, n = 122). Lower traffic exposure at birth showed a upward shift (decelerated shortening) in telomere ranking between birth and young adulthood (Fig. 3b). A doubling in distance in early-life residential distance to a major road was significantly associated with an upward shift in ranking (2.9; 95% CI 0.29 to 5.5; *P* = 0.04) (Fig. 4b). We observed no significant association of residential distance to a major road in young adulthood or in persons living at the same address their whole life (non-movers) with adult telomere length and telomere ranking. Finally, we ran a sensitivity analysis in movers to estimate the independence of early-life exposure from adulthood exposure and therefore combined both early-life and adulthood residential proximity to a major road in the same model along with the aforementioned covariates. This was confirmatory, showing that a doubling in distance in early-life residential distance to a major road was significantly associated with an upward shift in ranking (2.6; 95% CI 0.5 to 4.8; *P* = 0.02).

## Discussion

Persons of the same age vary greatly with regards to telomere length, and this variation is present from early life [35]. Here, we studied telomere tracking from birth to young adulthood (18–30 years). The combination of both telomere measurements at birth and in adulthood is unique and has not been previously reported. Telomere length at birth (placental tissue) and in adulthood (buccal cells) were positively correlated. Independent of telomere length at birth, a lower buccal telomere length was observed in adults who had higher residential exposure to traffic early in life. In addition, the rank changes from birth to adulthood showed a stronger downward shift in telomere ranking over life in those that had higher residential traffic exposure at the birth address, while traffic exposure at the residential address at adult age was not associated with telomere length. Our study has important implications; based on telomere measures at birth and in adulthood, we found that the inverse association between prenatal traffic-related exposure and telomere length is not only perceived at birth but lasts over the life course.

We show a positive association between placental telomere length at birth and buccal telomere length in adulthood, which emphasizes the importance of understanding factors that determine early-life telomere length. An experimental study in zebra finches showed that telomere length early in life is a strong predictor of lifespan [36].

Our findings between telomere length and traffic exposure are in line with recent reported associations between adult traffic exposure and telomere length in adulthood. In Belgium, residential annual average exposure to particles less than 2.5 μm in diameter (PM_2.5_) was associated with shorter telomere length, lower mtDNA content, and reduced *SIRT1* expression in peripheral blood leukocytes of the elderly [21]. In a cohort of 165 never-smoker elderly men, an IQR increase in annual black carbon exposure (0.25 μg/m^3^), a marker of traffic-related air pollution, was associated with an 8% decrease in leukocyte telomere length (95% CI –13% to –2%) [19]. Further, a study comparing traffic officers exposed to high levels of traffic pollutants with office workers showed shorter telomere length in traffic officers [20].

We assume that the underlying mechanism between traffic exposure and telomere length is oxidative stress and inflammation. Road traffic contributes largely to particulate matter pollution. Phagocytosis of these particles by alveolar macrophages in the lungs can result in oxidative stress and inflammation [37, 38]. However, traffic-related ultrafine particles can even translocate into the blood circulation and result in local effects [39]. The blood circulation of the mother can transport these particles to the placenta and particles with a diameter of less than 240 nm are able to cross the placental barrier, resulting in direct effects [40]. However, even larger particles, which are not able cross the barrier, can cause oxidative stress in the placenta and indirectly affect the fetus. Recently, it was shown that maternal exposure to particulate air pollution during pregnancy was associated with higher placental 3-nitrotyrosine levels, a biomarker of oxidative stress [41]. Telomeres are highly sensitive to oxidative stress due to their high guanine content and the inefficient repair system of single-strand breaks [42].

Besides oxidative stress, an underlying link between traffic exposure and telomere length may be maternal perceived stress. Previous studies [43–45] highlight the importance of stress when studying telomere length. It was found that women with the highest levels of perceived stress have, on average, shorter telomeres by the equivalent of at least one decade of additional aging compared to low stress women [43]. Further research showed that maternal psychological stress during pregnancy is associated with newborn telomere length [44] and that prenatal exposure to stress was significantly associated with shorter telomere length in young adulthood [45]. The association between buccal telomere length and traffic exposure remained significant after additional adjustment for cortisol. However, higher traffic-related exposure might also induce higher prenatal stress. A limitation of this study is that cortisol was not measured at birth.

Our findings suggest an inverse association between telomere attrition and initial telomere length, wherein attrition rate is more pronounced in newborns with longer placental telomeres at baseline. These results are consistent with previous reports of an inverse association between telomere attrition rate and initial telomere length in blood samples [12, 46, 47]. Three reasons for the inverse association between telomere attrition and initial telomere length have been proposed [12, 46, 47]. First, longer telomeres are more susceptible to oxidative stress due the sensitivity of the telomeric G triplet to oxidative damage [12]. Second, telomerase acts preferentially on short telomeres as a protection mechanism [47]. Third, it might be possible that the correlation between telomere attrition and baseline telomere length is largely, but not completely, explained by the statistical phenomena of regression to the mean [48]. In our analysis, we accounted for the phenomenon of regression towards the mean by adjusting for telomere length at baseline.

The present study should be interpreted in the context of its limitations. First, telomere length at birth and in young adulthood have been measured in different tissues and with slightly different methods. The rates of telomere shortening are similar in somatic tissues [49]. However, to avoid methodological issues due to difference in tissue and methods, we rather focused on the change in telomere ranking over this period. Second, telomere length in adulthood was not measured in blood but in buccal cells. Despite the absolute difference in telomere length between tissues, strong intra-individual correlations in telomere length have been observed between blood and buccal cells [50]. However, these results were not confirmed by other related studies [51]. In adults [49], and even at birth [52, 53], a high synchrony in telomere length between tissues is present and age-dependent telomere attrition during adulthood is very similar across different somatic tissues. A disadvantage of buccal cells is that poor oral hygiene or infection during sampling can alter oral cell composition [54]. Nevertheless, buccal cell telomeres may be more inert and less influenced by regulatory factors than white blood cells due to the different cell populations in blood [54]. Third, we do not have high resolution residential air pollution data of the pregnancy period. Indeed, the samples of this twin study were collected from 1969 to 1982 and no high resolution ambient air pollution data of this period are available. A limitation inherent to the use of a proxy marker for traffic exposure, i.e., residential distance to a major road, is that it does not take into account individual differences in the time spent at home and in other environments. However, the proxy of residential distance to a major road is currently still used in environmental epidemiology [55–57] and we recently showed that it correlates with the black carbon load in children’s urine [58]. Fourth, residential distance to a major road at birth and in adulthood were correlated (r = 0.32; *P* < 0.001). Therefore, to detangle between early and late exposure we cannot completely rule out residual confounding. Nevertheless, a sensitivity analysis, in which both early- and later-life exposure were combined in the same model, confirms the stronger impact of early-life exposure.

## Conclusions

To our knowledge, this is the first longitudinal study measuring telomere length at birth and in adulthood. We showed a correlation between telomere length in placental tissue and in buccal cells in young adulthood. In addition, our results emphasize the importance of the early-life environment on adult telomere length. We noted that traffic-related exposure in early life is not only associated with shorter telomere length in placental tissue but also shorter telomere length in young adulthood. As telomere length is a proposed measure of biological aging, these results suggest that environmental exposure in early life may have implications for health outcomes later in life and that age-related diseases may have their origin in the early-life environment.

## Additional file

Additional file 1: Figure S1. Distance to the nearest major road in association with (A) telomere length in buccal cells and (B) change in telomere length ranking between birth and adulthood. Adjusted for newborn sex, birth weight, gestational age, zygosity and chorionicity, parental education level, maternal smoking during pregnancy, maternal age, adult age, smoking in adulthood, gamma-glutamyl transferase in fasting blood in adulthood (as an index for alcohol consumption), 24-h urinary total cortisol, and telomere length in placental tissue at birth. Vertical lines denote 95% confidence intervals. *Indicates significant (*P* < 0.05) change in buccal telomere length in adulthood or change in telomere ranking. Effect size for a two-fold increase in distance from residence to major road in early/adult life (based on a model with log distance) in movers (n = 109) or in distance from residence to major road during whole life in non-movers (n = 57). (DOCX 196 kb)

## Abbreviations

CI: confidence interval
IQR: interquartile range
SD: standard deviation
T/S ratio: telomere to single copy gene ratio
