# Supplementary material for: Telomere tracking from birth to adulthood and residential traffic exposure
Source: BMC Med. 2017 Nov 21;15:205. doi: 10.1186/s12916-017-0964-8 (PMC5697215; doi:10.1186/s12916-017-0964-8)
Supplement: Additional file 1: Figure S1. — Distance to the nearest major road in association with (A) telomere length in buccal cells and (B) change in telomere length ranking between birth and adulthood. Adjusted for newborn sex, birth weight, gestational age, zygosity and chorionicity, parental education level, maternal smoking during pregnancy, maternal age, adult age, smoking in adulthood, gamma-glutamyl transferase in fasting blood in adulthood (as an index for alcohol consumption), 24-h urinary total cortisol, and telomere length in placental tissue at birth. Vertical lines denote 95% confidence intervals. *Indicates significant (P < 0.05) change in buccal telomere length in adulthood or change in telomere ranking. Effect size for a two-fold increase in distance from residence to major road in early/adult life (based on a model with log distance) in movers (n = 109) or in distance from residence to major road during whole life in non-movers (n = 57). (DOCX 196 kb) [file 12916_2017_964_MOESM1_ESM.docx]

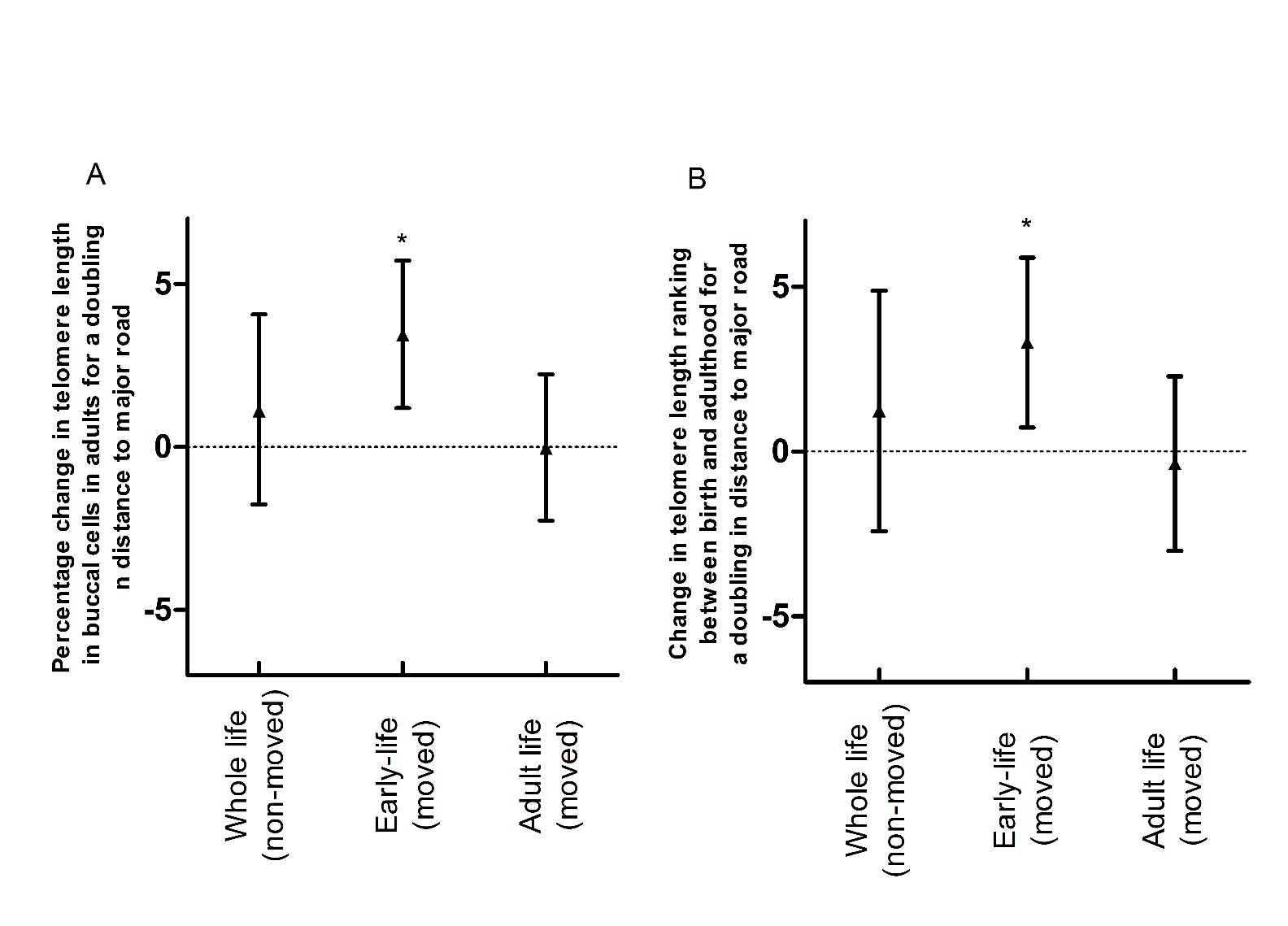


**Supplement figure 1** Distance to the nearest major road in association with A) telomere length in buccal cells and B) change in telomere length ranking between birth and adulthood. Adjusted for newborn sex, birth weight, gestational age, zygosity-chorionicity, parental education level, maternal smoking during pregnancy, maternal age, adult age, smoking in adulthood, gamma-glutamyl transferase in fasting blood in adulthood (as an index for alcohol consumption), 24-h urinary total cortisol, and telomere length in placental tissue at birth. Vertical lines denote 95% confidence intervals. *indicates significant (*P* < 0.05) change in buccal telomere length in adulthood or change in telomere ranking. Effect size for a twofold increase in distance from residence to major road in early/adult life (based on a model with log distance) in movers (n=109) or in distance from residence to major road during whole life in non-movers (n=57).
